# Geographic variation in maternal investment and trade-offs between egg size and clutch size in an endemic toad of the Qinghai-Tibet Plateau

**DOI:** 10.1038/s41598-020-63635-y

**Published:** 2020-04-22

**Authors:** Tong Lei Yu, Yao Hui Deng

**Affiliations:** Department of Biology, College of Life Science, Xinyang Normal University, Xinyang, SD 464000 China

**Keywords:** Evolutionary ecology, Evolutionary theory

## Abstract

Life history theory predicts that animals often produce fewer offspring of larger size and indicate a stronger trade-off between the number and size of offspring to cope with increasing environmental stress. In order to evaluate this prediction, we tested the life history characteristics of *Bufo minshanicus* at eight different altitudes on the eastern Tibetan Plateau, China. Our results revealed a positive correlation between female SVL and clutch size or egg size, revealing that larger females produce more and larger eggs. However, high-altitude toads seem to favor more offspring and smaller egg sizes when removing the effect of female SVL, which is counter to theoretical predictions. In addition, there was an overall significantly negative relationship between egg size and clutch size, indicative of a trade-off between egg size and fecundity. Therefore, we suggest that higher fecundity, rather than larger egg size, is a more effective reproductive strategy for this species of anuran living at high-altitude environments.

## Introduction

Life-history theory suggests that females should be able to optimize their allocation of resources between current breeding efforts and future survival^1^. Higher altitudes can be stressful environments because temperatures are colder, seasonality is greater, and food availability can fluctuate greatly. For example, there are negative correlations between temperature and altitude^2^, with rainfall and altitude found to be positively correlated^3^. High-altitude females are expected to select for producing larger eggs^2–6^ because larger eggs can lead to larger initial sizes, faster rates of growth, faster developmental rates, or both^7–12^. Yet, a significant increase in egg size may prolong the duration of the embryonic period, which results in increasing total mortality^13^. In addition, individuals that metamorphose at larger size may be more easily discovered by predators, thus Rollinson and Hutchings (2010)^14^ suggest that bigger is not always better. However, egg or hatching size may not affect mass at metamorphosis and survival because other ecological factors, such as temperature, density and competition, food level and quality, predation, or a combination of these, can alter the growth and developmental rate of each life stage of amphibians^9,15–18^.

The number of offspring produced, and their size, are fundamental life history traits because they are intimately linked with fitness and therefore population viability^19^. The trade-off between offspring number and size suggests that selection will produce more small eggs or abundant larvae under scarce resources and stressful environments, which is known as *r*-selection^9^. The advantage associated with producing smaller eggs is due to the corresponding increase in fecundity^9^. Conversely, selection will prefer to produce larger eggs or more robust larvae under abundant essential resources and good environmental conditions, which is regarded as *K*-selection^5,20^. However, amphibians respond to varying environments by changing egg size or a trade-off between clutch size and egg size^18,21^.

At higher altitudes, many species have relatively short breeding seasons and active cycles, resulting in slower growth and usually larger size than the same species from warmer areas^16,22,23^. To date, however, we have a very poor understanding of how maternal investment varies geographically in a high altitude species in response to selective pressure. Thus, we investigated altitudinal variations in *Bufo minshanicus* life history traits, focusing on a trade-off between egg size and clutch size. So far, the taxonomic status of *B. minshanicus* has been controversial for over half a century. Fu *et al*. (2005)^24^ suggested *B. minshanicus* and *Bufo andrewsi* were treated as a single species^25^, *Bufo gargarizans*, without subspecies division. However, Fei and Ye (2000)^26^ treated *B. minshanicus* as a subspecies of *B. gargarizans*. In this study, we defined *B. minshanicus* as a valid species. *Bufo minshanicus* is a species endemic to high altitude regions of the eastern Tibetan Plateau in China. They are widely distributed in forests, fields, and open alpine marshes from 2500 to 3700 meters (m) altitude and one of the few amphibian species to live at such high altitudes globally (Fei and Ye 2001). Geographically widespread species that occupy many thermal environments provide testable models for understanding the evolution of life-history responses to altitude. As a typical explosive breeder^27^, *Bufo minshanicus* exhibits a short breeding season (5–18 days). Females are larger than males^28^ and clutch size increases with increasing female SVL^29,30^. Low latitude populations in the eastern Qinghai province select deep swamps as hibernation site, while high latitude populations in the southern Gansu province prefer rabbit or otter holes as hibernating sites. Studies across altitudes provide a good approach to understand life-history evolution in stressful environments. Here, we predict that altitude would be (1) negatively correlated with clutch size, (2) positively associated with egg size, and (3) positively correlated with female SVL. We also tested the prediction that (4) there is a trade-off between egg size and clutch size.

## Materials and methods

### Data collection

We collected *Bufo minshanicus* individuals from eight populations (all collected individuals from the same pond during the breeding period were defined as a population, at elevations ranging from 2506 to 3478 m) along the eastern Tibetan Plateau, China. At these sites, annual mean air temperatures decrease with increasing altitude or decreasing latitude, whereas annual total precipitation increases with increasing altitude or decreasing latitude (Table 1). The Gahai-Zecha National Nature Reserve Management Bureau approved this project (approval number GHZCRMB/03-212014), and gave permission for fieldwork. All animals were collected under the guidelines for animal care in China. Handling and processing of toads followed approved protocols from the Animal Scientific Procedures Act 1988 by the State Department of China. A total of 683 amplectant pairs were collected during the peak period of breeding activity from 2009 to 2015 (Table 1; Dataset 1). We transported them to our field lab located close to breeding sites. Each amplexed pair was put into a plastic container (2-liter) until the eggs were deposited. After counting eggs for each clutch and measuring SVL of both sexes to the nearest 1 mm using a ruler, the eggs and adults were returned to the breeding pond.

**Table 1:** Study site details, including altitude, latitude and sample sizes and clutch attributes for 8 high-altitude *Bufo minshanicus* populations in the Tibetan Plateau. Values represent mean ± SE (*n*) for each measure. *n* = number of individuals.

Altitude (m)	Latitude (degrees)	Precipitation (mm)	Temperature (°C)	Clutch size	Egg size (mm)	Clutch volume (mm^3^)	Mean body size (mm)	Collection years
2506	36.57	576	6.1	4465 ± 171 (62)	1.97 ± 0.02 (97)	19318.2 ± 895.0 (62)	85.1 ± 0.8 (52)	2014, 2015
2593	36.67	540	5.7	4024 ± 98 (155)	2.02 ± 0.01 (156)	17465.3 ± 442.0 (155)	83.9 ± 0.4 (155)	2012, 2014, 2015
3027	34.48	584	4.5	2354 ± 49 (210)	2.05 ± 0.01 (210)	10756.7 ± 289.0 (210)	73.0 ± 0.3 (209)	2013, 2014, 2015
3211	34.38	586	3.9	2578 ± 273 (3)	1.86 ± 0.04 (18)	6260.5 ± 1530.3 (3)	73.3 ± 1.5 (3)	2014
3230	34.10	600	4.1	2602 ± 109 (49)	2.05 ± 0.02 (49)	11767.6 ± 504.5 (49)	76.2 ± 1.5 (9)	2015
3443	34.30	590	3.4	3225 ± 84 (99)	1.93 ± 0.01 (99)	12346.5 ± 402.0 (99)	76.3 ± 0.5 (98)	2009, 2013, 2014, 2015
3477	34.20	591	3.3	3563 ± 115 (103)	1.90 ± 0.01(115)	12653.3 ± 374.1 (103)	75.6 ± 0.4 (103)	2009, 2013, 2014
3478	34.23	595	3.3	4215 ± 167 (52)	1.82 ± 0.02 (52)	13429.5 ± 628.1 (52)	82.0 ± 0.7 (52)	2015

About 100 eggs were randomly selected from each clutch and placed on a glass plate with calipers to take a digital image. We obtained measurements of individual egg sizes (n = 20–30; ±0.01 mm) excluding the jelly using a tpsdig2 computer software. Clutch volume was calculated by multiplying the number of oviposited eggs with the volume of each egg (*Vs* = 4/3πr^3^, where r is the radius). We obtained average ambient temperatures and precipitation from 2009 to 2015 from the Chinese Meteorological Administration (http://www.cma.gov.cn) and the Gansu Gahai-Zecha National Nature Reserve Management Bureau.

### Statistical analyses

Clutch size, egg size and clutch volume were analyzed using general linear mixed models (GLMMs) where population was treated as a random factor, and altitude and latitude as covariates. Egg size and clutch size were used as covariates of one another when testing for a trade-off between them. To investigate variation in body size of females, we used GLMMs where female SVL was the dependent variable, latitude and altitude as covariates, and population as a random factor.

In the clutch-by-female analyses, we treated clutch size, egg size and clutch volume as the dependent variable and female SVL, latitude and altitude as covariates. Finally, to investigate the trade-off between egg size and clutch size, we used egg size or clutch size as the dependent variable, the other as the independent variable, and then the other way around. Prior to analyses, we used log-transformed raw data on body size, clutch size, egg size and clutch volume. Meanwhile, we removed one population (3211 m) because only 3 female toads were collected. The analyses were started with a full model, with all possible interactions, and then removing non-significant interactions (*P* > 0.1, for details, see Räsänen *et al*. 2008^21^) because interaction effects in many cases may be statistically weaker, but biologically important. Thus, in this case, it would be important to report the effect sizes rather than actual *P* values. For the *R*^2^ values, as we used GLMM with both fixed effects and random factors, it thus was needed to provide the *R*^2^ marginal (*R*^2^_m_) as the amount of variation explained by fixed factors, and *R*^2^ conditional (*R*^2^_c_) as amount of variation explained by both fixed and random factors. All the analyses were done with IBM SPSS Statistics 20.0 (IBM Corp, Armonk, NY, USA).

## Results

### Clutch data

**Figure 1:**
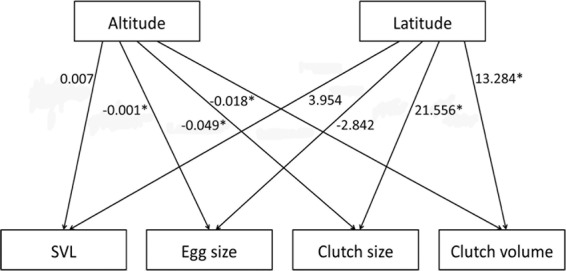
Regression coefficients (slope, 100b) for effects of altitude and latitude on maternal investment parameters (egg size, clutch size, and clutch volume [total reproductive output]) and female body size in 7 populations. *P < 0.05; **P < 0.01.

Egg size decreased with increasing altitude (*F*_1, 3.863_ = 8.397, *P* = 0.046), but not latitude (*F*_1, 3.879_ = 5.357, *P* = 0.084, *R*^2^_m_ = 0.17, *R*^2^_c_ = 0.29, Fig. 1), providing weak evidence for correlation between egg sizes and altitudes. Clutch size increased with increasing altitude and latitude (altitude, *F*_1, 3.879_ = 10.035, *P* = 0.035; latitude, *F*_1, 3.856_ = 15.186, *P* = 0.019, *R*^2^_m_ = 0.30, *R*^2^_c_ = 0.41, Tables 1, 2, Fig. 1). Clutch volume increased with increasing latitude (*F*_1, 3.620_ = 14.910, *P* = 0.022,), but not altitude (*F*_1, 3.702_ = 3.636, *P* = 0.135, *R*^2^_m_ = 0.26, *R*^2^_c_ = 0.29).

**Table 2:** Analyses of covariance of egg size, clutch size, and total reproductive output (clutch volume) in females from 7 high-altitude *Bufo minshanicus* populations. **P < 0.001,*P < 0.05.

Source of variation	Random			Fixed	
	VAR	SE	*Z*	df	*F*
**Egg size**					
Residuals	0.000894	0.000046	19.633**		
Population	0.000148	0.000113	1.308		
Latitude				3.846	5.357
Altitude				3.863	8.397*
**Clutch size**					
Residuals	0.017091	0.000899	19.013**		
Population	0.003014	0.002295	1.313		
Latitude				3.856	15.186*
Altitude				3.879	10.035*
**Clutch volume**					
Residuals	0.021734	0.001144	19.001**		
Population	0.001019	0.000906	1.125		
Latitude				3.620	14.910*
Altitude				3.702	3.636

There was a negative association between clutch size and egg size, indicating a population-level trade-off between fecundity and egg size (and vice versa; clutch size, b ± SE = −0.03062 ± 0.00816, *F*_1, 723.428_ = 14.070, *P* < 0.001, *R*^2^_m_ = 0.22, *R*^2^_c_ = 0.28; egg size, b ± SE = −0.61211 ± 0.16551, *F*_1, 725.937_ = 13.678, *P* < 0.001, *R*^2^_m_ = 0.32, *R*^2^_c_ = 0.41).

### Clutch-by-female data

Female body size tended to increase with increasing latitude and altitude, but the difference was not significant (latitude, b ± SE = 0.03954 ± 0.01590, *F*_1, 4.088_ = 6.185, *P* = 0.066; altitude, b ± SE = 0.00007 ± 0.00004, *F*_1, 4.006_ = 2.242, *P* = 0.209, *R*^2^_m_ = 0.35, *R*^2^_c_ = 0.52, Table 1, Fig. 1).

**Figure 2:**
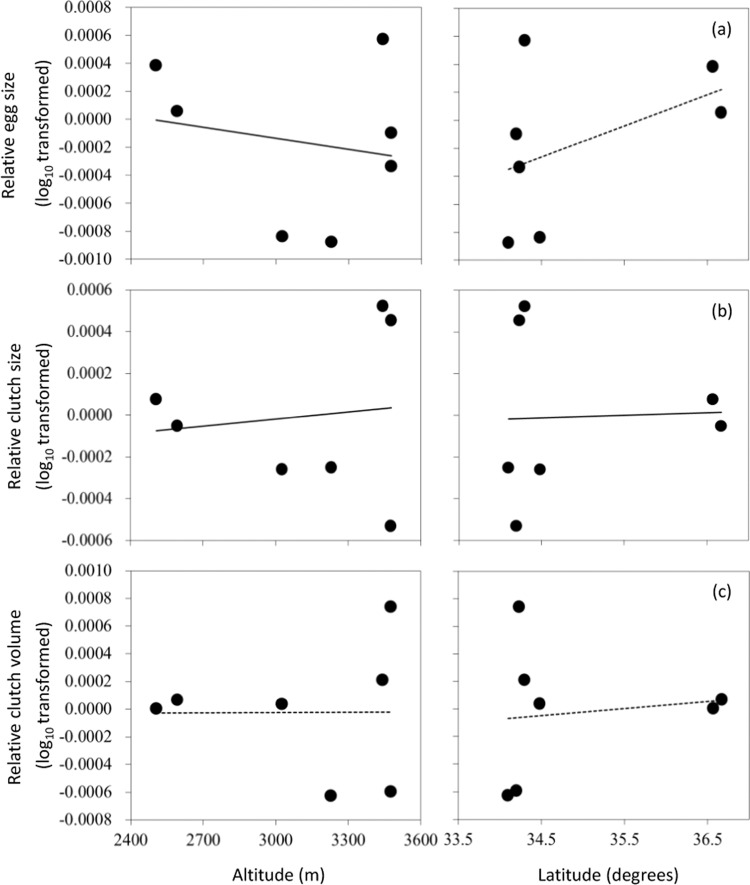
Relationship between altitude or latitude and egg size **(a**), clutch size (**b**) and total clutch volume (**c**) in 7 *Bufo minshanicus* populations. Solid lines: *P* < 0.05; dashed line: *P* > 0.05. Data points are population means. Relative log_10_ was generated from regression of log_10_ (egg size) or log_10_ (clutch size) or log_10_ (total clutch volume) on log_10_ (female body size). Statistical details are as in Table 3.

GLMMs revealed that egg size, clutch size, and clutch volume were associated with female body size (egg size, *F*_1, 665.984_ = 4.349, *P* = 0.037; clutch size, *F*_1, 316.444_ = 284.267, *P* < 0.001; clutch volume, *F*_1, 597.455_ = 254.250, *P* < 0.001, Fig. 2). When removing the effect of female size, egg size decreased significantly with increasing altitude (*F*_1, 3.913_ = 9.586, *P* = 0.037), but not latitude (*F*_1, 4.138_ = 4.618, *P* = 0.096, *R*^2^_m_ = 0.20, *R*^2^_c_ = 0.30). Clutch volume did not significantly vary with altitude (*F*_1, 1.428_ = 0.146, *P* = 0.752) or latitude (*F*_1, 1.526_ = 0.781, *P* = 0.494, *R*^2^_m_ = 0.47, *R*^2^_c_ = 0.49), but clutch size increased significantly with increasing altitude (*F*_1, 3.258_ = 12.753 *P* = 0.033) and latitude (*F*_1, 3.247_ = 15.689, *P* = 0.025). The interaction between altitude and latitude was significant (*F*_1, 3.292_ = 11.899, *P* = 0.035, *R*^2^_m_ = 0.53, *R*^2^_c_ = 0.55, Table 3, Fig. 2) suggesting that clutch size varied inversely with altitude at higher latitudes.

**Table 3:** Analyses of covariance of egg size, clutch size, and total reproductive output (clutch volume) in females from 7 high-altitude *Bufo minshanicus* populations in a model including female size. **P < 0.001,*P < 0.05.

Source of variation	Random			Fixed			
	VAR	SE	*Z*	*b*	SE	df	*F*
**Egg size**							
Residuals	0.000822	0.000045	18.320**				
Population	0.000106	0.000086	1.227				
Female size				0.088019	0.042208	665.984	4.349^*^
Latitude				0.023973	0.011156	4.138	4.618
Altitude				−0.000094	0.000030	3.913	9.586^*^
**Clutch size**							
Residuals	0.012357	0.000677	18.264**				
Population	0.000013	0.000147	0.087				
Female size				2.663521	0.157977	316.444	284.267^**^
Latitude				0.696571	0.175863	3.247	15.689^*^
Altitude				0.008521	0.002386	3.258	12.753^*^
Latitude × altitude				−0.000242	0.000070	3.292	11.899^*^
**Clutch volume**							
Residuals	0.016005	0.000877	18.254**				
Population	0.000752	0.000736	1.022				
Female size				2.945306	0.184714	597.455	254.250**
Latitude				0.021616	0.032701	3.586	0.437
Altitude				0.000002	0.000088	3.344	0.001

Clutch size was negatively correlated with egg size when accounting for female size within each population, but the correlations for only two populations were significant (both *r* > −0.24, *P* < 0.003, Table 1). The clutch-by-female data further showed an overall significant negative relationship between egg and clutch size (and vice versa; clutch size, b ± SE = −0.05014 ± 0.00979, *F*_1, 667.605_ = 26.223, *P* < 0.001, *R*^2^_m_ = 0.24, *R*^2^_c_ = 0.32; egg size, b ± SE = −0.75571 ± 0.14644, *F*_1, 659.540_ = 26.633, *P* < 0.001, *R*^2^_m_ = 0.56, *R*^2^_c_ = 0.57), indicative of a trade-off between egg size and clutch size. The slopes of egg size decreased with increasing altitude and latitude, but the difference was not significant (altitude × egg size, *F*_1, 661.082_ = 1.677, *P* = 0.196; latitude × egg size, *F*_1, 666.610_ = 1.368, *P* = 0.243).

## Discussion

In order to maximize fitness, life history traits vary under different environmental pressures^31,32^. In this study, our results showed that maternal investment strategies differ among altitudes. When the effect of body size is removed, the clutch data across seven *B. minshanicus* populations indicated that females are more fecund but produce smaller egg sizes at higher altitudes than at lower altitudes. The clutch-by-female size further showed a population-level trade-off between fecundity and egg size.

However, if the outlier population (at 3211 m) was not removed, the clutch data across eight *B. minshanicus* populations indicated that egg size and clutch volume tend to decrease or increase with increasing altitude (*F*_1, 4.409_ = 4.19, *P* = 0.104) or latitude (*F*_1, 0.926_ = 8.47, *P* = 0.218), while female SVL increased with increasing latitude (*F*_1, 4.784_ = 7.84, *P* = 0.040). Since those results were not consistent among populations in this study, additional research may be needed with more intensive sampling efforts along more geographical gradients.

In this study, female SVL was not significantly positive correlated with latitude or altitude. This result was not consistent with previous evidence considering six populations^28^. The shorter seasonal period of activity, weaker digestion and lower energy of prey may limit larger body size for appearing at high altitudes or latitudes. Moreover, females living in higher-altitudes must store enough energy during the short activity period in order to survive the harsh and extended winter, which may become more difficult for larger individuals. Therefore, Chen *et al*. (2013)^4^ suggested that larger females at high altitudes may be costly.

Life history theory suggests that organisms should allocate the limited energy between growth and development, thus creating a trade-off between growth and development^1^. The clutch data across seven *B. minshanicus* populations revealed that clutch attributes (clutch size, egg size, and clutch volume) were positively correlated with female size, revealing that larger females produce more eggs, larger eggs and increase reproductive effort. In fact, most amphibians and reptiles, in general, exhibit this relationship between body size and reproductive output. Thus, it is attributed to positive fecundity selection, where female body size is larger for larger clutches^33^. Like most ectotherms with indeterminate growth, female *B. minshanicus* fit the von Bertalanffy’s model^28^, which describes rapid somatic growth for earlier stages followed by slower growth thereafter. This implies that a larger fraction of energy would be devoted to reproduction as individuals become older^34,35^, thus resulting in age-specific reproductive output.

In some anurans, larger clutches in high-altitude or latitude populations are due to the larger size of high-altitude or latitude females^16,22,36,37^. When removing the effect of female body size, females at high altitudes produce relatively fewer clutches, but larger eggs than conspecifics from low altitudes^5,16,23,38^. However, in this study, after controlling for the effect of female size, higher altitude females had higher fecundity and smaller egg sizes with similar reproductive effort than lower altitudes females, thus the endemic plateau toad has evolved a different breeding strategy. This result was consistent with a previous study^39^, which showed that the phrynosomatid lizard *Phrynosoma cornutum* had smaller females at higher latitudes that produced larger clutches but similarly sized eggs when compared to larger females at lower latitudes and removing the effect of female size. It is well known that rainfall can directly affect the abundance of food, and may indirectly affect fecundity by reducing fat stores, which is necessary for development of a holistic egg number^40^. Females may have more resources for reproduction and clutch production if food availability is higher and physiological stress is lower. Interestingly, in this study, rainfall increases with increasing altitude, indicating higher food availability at higher altitudes. In this case, we speculated that resource may not limit clutch production at higher altitudes. Meanwhile, this species selects rabbit or otter holes as hiding and hibernating sites in the field. In this case, they reduce chances of predation and protect against extreme temperature fluctuations, especially in summer and winter. Additionally, disease and predation tend to decline when increasing altitude^41^. Thus, we speculate that this species does not allocate as much energy to survive the harsh and extended winter, and suggest females living at high altitudes likely allocate more energy for producing clutches. Similarly, Dobzhansky (1950)^42^ suggested that *r*-selection strategies were more likely to appear in changing environments (e.g. temperate and high altitude regions), whereas *K*-selection more likely to be found in relatively stable situations (e.g. tropics). As a consequence, larger clutch size is regarded as an attribute that populations have evolved through time as an adaptation to their general environment and their ecological niche^43,44^.

Life-history theory emphasizes that high-altitude females are expected to select for the production larger eggs^2–6^ because larger eggs or offspring will lead to larger initial sizes, faster growth speeds, higher developmental speeds, or both^7–12^. However, our results showed egg size across seven *B. minshanicus* populations decreased with increasing altitude, which provide support for the notion that life-history strategies respond to environmental conditions. Similarly, in some instances smaller eggs are better^45^ or some studies report no significant difference between egg size and altitude^46,47^. Other studies found that some species with small eggs will increase the number of offspring^48^, especially in the uncertain larval environment. Additionally, small eggs have a relatively large surface-to-volume ratio and require relatively little oxygen during embryonic development^49^. In this case, this animal may better be able to adapt to the anoxic environment of the Tibetan Plateau.

Although some studies suggest that trade-offs between clutch size and egg size in amphibians seldom occurs^4,50–53^, some previous findings^5,9,54,55^ support the existence of a trade-off. In this study, although intra-population-level trade-off between fecundity and egg size was uncommon, there was a significant inter-population-level trade-off between fecundity and egg size, suggesting that females producing greater numbers will produce smaller eggs to compensate for the energy put into the greater numbers, which are consistent with the general principle of MacArthur and Wilson’s theory of *r*-versus *K*-selection in populations^56,57^. Moreover, higher altitudes influenced investment in egg size, as suggested by the fact that there were stronger negative effects on egg size than on clutch size and reproductive output. Our data also showed that the strength of the trade-off between clutch size and egg size was not different among altitudes and latitudes. However, theoretical^53,58^ and empirical^9,21,54^ studies showed that harsh environments promote larger maternal investment in per-individual offspring to improve survival of individual offspring. For *B. minshanicus*, at higher altitudes, the advantage associated with producing smaller eggs because of increasing fecundity, which potentially maximizes the number of offspring surviving to reproduction^10^. Therefore, different environmental pressures will lead to geographical variation in life history traits aimed at maximizing fitness^31,32^. *Bufo minshanicus* have evolved a different life history strategy to adapt their environments, which may be inconsistent with our expectations. On the Tibetan Plateau, it appears as though more fecundity and smaller egg size are traits that enhance fitness for this animal.

## Supplementary information

Supplementary information
